# Relationships between personality traits and attitudes toward the sense of smell

**DOI:** 10.3389/fpsyg.2013.00901

**Published:** 2013-11-28

**Authors:** Han-Seok Seo, Suji Lee, Sungeun Cho

**Affiliations:** Department of Food Science, University of ArkansasFayetteville, AR, USA

**Keywords:** attitude toward sense of smell, personality traits, gender, the Eysenck Personality Questionnaire-Revised, lie-scale

## Abstract

Olfactory perception appears to be linked to personality traits. This study aimed to determine whether personality traits influence human attitudes toward sense of smell. Two-hundred participants’ attitudes toward their senses of smell and their personality traits were measured using two self-administered questionnaires: the Importance of Olfaction Questionnaire and the Eysenck Personality Questionnaire-Revised. Demographics and olfactory function were also assessed using a self-administered questionnaire. Gender-induced differences were present in attitudes toward sense of smell. Women participants were more dependent than men participants on olfactory cues for daily decision-making. In addition, as participants evaluated their own olfactory functions more positively, they relied more on olfactory information in everyday life. To determine a relationship between personality traits and attitudes toward sense of smell, Spearman partial correlation analyses were conducted, with controlling the factors that might influence attitudes with respect to sense of smell (i.e., gender and self-awareness of olfactory function) as covariates. Participants who scored high on the lie-scale (i.e., socially desirable and faking good), tended to use olfactory cues for daily decision-making related both to social communication and product purchase. In conclusion, our findings demonstrate a significant association between personality traits and attitudes toward sense of smell.

## INTRODUCTION

Despite its important role, relatively little attention has been paid to the sense of smell compared to other senses (e.g., vision and hearing). The sense of smell is mainly associated with eating behavior, awareness of environmental hazard, and social communication (for a review, see Stevenson, 2010). Olfactory function influences appetite (De Jong et al., 1999), food perception and palatability (Aschenbrenner et al., 2008; Seo and Hummel, 2009; Novakova et al., 2012; Schubert et al., 2012), and food-related social behavior (Aschenbrenner et al., 2008). For example, people with olfactory impairment appear to be more exposed to risks of unbalanced nutritional status (Duffy et al., 1995; Schiffman and Graham, 2000) and poor food intake (Aschenbrenner et al., 2008), although these findings have not been consistently observed in previous studies (De Jong et al., 1999; Schubert et al., 2012; Smoliner et al., 2013). In addition, a sense of smell can detect not only microbial risks such as feces, decay, and spoilage (Stevenson, 2010), but also non-microbial threats such as gas leaks and smoke (Miwa et al., 2001; Santos et al., 2004; Croy et al., 2012). Finally, the major histocompatibility complex (MHC) genotype and body odors can play a critical role in mate selection, not only by avoiding inbreeding, but also by detecting fit partners (Wedekind et al., 1995; Gangestad and Thornhill, 1998; Herz and Inzlicht, 2002; Croy et al., 2013; for a review, see Yamazaki and Beauchamp, 2007; Stevenson, 2010). For example, women students rated body odors of T-shirts worn by men different from themselves with respect to MHC alleles significantly more pleasant than body odors of T-shirts worn by men with similar MHC alleles (Wedekind et al., 1995). Olfactory signals can also deliver individual identity (Olsson et al., 2006; Lundström et al., 2008), emotional states (Chen and Haviland-Jones, 2000; Prehn-Kristensen et al., 2009; Croy et al., 2011a), age-related information (Mitro et al., 2012), and sexual interests (Croy et al., 2013). Croy et al. (2013) demonstrated an interesting relationship between sense of smell and sexual relationships in people diagnosed with isolated congenital anosmia. Men born without a sense of smell reported significantly fewer sexual relationships compared to age-matched healthy men. Also, women born without a sense of smell appeared to feel less secure about sexual partnership compared to healthy women in a control group.

Although the sense of smell plays a significant role in modulating eating behavior, hazard detection, and social communication (Stevenson, 2010), people’s attitudes toward sense of smell vary as a function of olfactory performance (Frasnelli and Hummel, 2005; Shu et al., 2011), gender (Frasnelli and Hummel, 2005; Ferdenzi et al., 2008; Havlicek et al., 2008; Croy et al., 2010; Seo et al., 2011), and culture (Schleidt et al., 1981; Schaal et al., 1997; Ferdenzi et al., 2008; Seo et al., 2011). For example, patients with olfactory impairments tend to complain more strongly about their decreased quality of life than people with normal olfactory function (Frasnelli and Hummel, 2005). Furthermore, women patients consider olfactory impairment-decreased quality of life more negatively than do men patients (Frasnelli and Hummel, 2005). Gender-induced difference in attitudes toward olfaction is also observed in people with a normal sense of smell (Ferdenzi et al., 2008; Havlicek et al., 2008; Croy et al., 2010). It seems that women are more attentive than men to olfactory cues (Ferdenzi et al., 2008; Havlicek et al., 2008; Croy et al., 2010; Seo et al., 2011).

Personality is another potential factor in modulating olfactory perception (Koelega, 1970, 1994; Filsinger et al., 1987; Pause et al., 1998; Larsson et al., 2000; Chen and Dalton, 2005; Havlíček et al., 2012;; La Buissonnière-Ariza et al., 2013). Earlier research demonstrated plausible relationships between olfactory sensitivity and personality traits such as extraversion/introversion; the results, however, are controversial. Koelega (1970) reported that olfactory sensitivity was positively correlated with degree of extraversion but not with degree of neuroticism. In contrast, another study by Herberner et al. (1989) demonstrated that, in comparison to extremely sociable participants, extremely shy participants were significantly more sensitive to odors. Furthermore, several studies reported no significant relationship between olfactory sensitivity and extraversion/introversion (Filsinger et al., 1987; Koelega, 1994; Pause et al., 1998; Larsson et al., 2000; Havlíček et al., 2012). Pause et al. (1998) found that neuroticism, when compared to extraversion, has a stronger impact in determination of olfactory sensitivity. Havlïvc (2012) also reported that olfactory sensitivity correlated with neuroticism, but not with other personality traits such as extraversion, openness, and agreeability (but see also Croy et al., 2011b). In addition, personality traits may alter a participant’s ability to identify odors (Larsson et al., 2000; Havlíček et al., 2012). For example, participants who scored high in neuroticism (i.e., more emotional and anxious) identified odors more correctly (Larsson et al., 2000). In contrast, participants with high degrees of impulsiveness and assertiveness identified odors less correctly (Larsson et al., 2000). A recent study conducted by Havlïvc (2012) found a significant correlation between participants’ anxiety traits (a neuroticism facet) and their ability to discriminate odors. That is, as participants were more anxious, they discriminated odors more correctly. Finally, personality modulates participants’ reaction speed with respect to olfactory cues (Chen and Dalton, 2005). Chen and Dalton (2005) demonstrated that both neurotic and anxious men detected pleasant/unpleasant odors more quickly than emotionally neutral odors, while stable and calm men detected both odors equally quickly (i.e., no significant differences in reaction time to both pleasant/unpleasant and neutral odors). In a more recent study, La Buissonnière-Ariza et al. (2013) compared response times of both high- and low-trait anxiety adults to pleasant- and unpleasant-smelling food odors (i.e., strawberry and fish odors, respectively). Similarly to previous findings of Chen and Dalton (2005), they found that, regardless of whether odors were pleasant or unpleasant, highly anxious individuals detected odors more quickly than did less anxious ones.

Likewise, earlier studies have highlighted the modulatory effects of personality traits on olfactory perceptions such as odor sensitivity, discrimination, and identification. In addition, previous research has demonstrated that people’s attitudes toward sense of smell can vary as a function of olfactory performance (Frasnelli and Hummel, 2005; Shu et al., 2011). Given the two ideas that (1) personality traits influence olfactory performance and (2) olfactory performance appears to be closely related to attitudes toward olfaction, we hypothesized that personality traits could be related to attitudes toward sense of smell. Up to now, little has been known about a potential connection between personality traits and attitudes toward sense of smell. To build on previous findings, this study has aimed to determine whether human attitudes toward sense of smell can be related to personality traits.

## MATERIALS AND METHODS

This study was conducted in conformance with the Declaration of Helsinki for studies on human subjects. The protocol was approved by the University Institutional Review Board of the University of Arkansas (Fayetteville, AR, USA).

### PARTICIPANTS

A total number of 207 volunteers (73 men and 134 women) representing an age range of 18–73 years [mean age ± standard deviation (SD) = 39 ± 14 years] took part in this study. Data from seven volunteers (four men and three women) who had either clinical histories of major diseases (e.g., diabetes and cancer) or olfactory impairment were discarded. The olfactory impairment was determined based on results obtained through a “Sniffin’ Sticks” screening test (Burghart Instruments, Wedel, Germany; for details, see Hummel et al., 2001). Accordingly, data from a total of 200 respondents (69 men and 131 women) were analyzed. **Table 1** shows the demographic details of the respondents. The experimental procedure was thoroughly explained to all participants and a written informed consent was obtained from each prior to participation.

**Table 1:** Participants’ demographic profiles and self-ratings of olfactory function.

			(*N* = 200)
Categories	Subcategories	Frequency	%
Gender	Men	69	34.5
	Women	131	65.5
Age group	18–24 years	27	13.5
	25–44 years	107	53.5
	45–64 years	59	29.5
	65 years and over	7	3.5
Body mass index	Underweight (less than 18.5)	6	3.0
	Normal weight (18.5–24.9)	77	38.5
	Overweight (25.0–29.9)	46	23.0
	Obese (more than 30.0)	70	35.0
Ethnicity background	Caucasian	193	96.5
	African-American	1	0.5
	Asian	6	3.0
Annual household income	Under $15,000	29	14.5
	$15,000 to $34,999	46	23.0
	$35,000 to $54,999	36	18.0
	$55,000 to $74,999	31	15.5
	$75,000 to $94,999	27	13.5
	More than $95,000	31	15.5
Education level	High school	62	31.0
	2-year college	28	14.0
	4-year college	62	31.0
	Graduate school	48	24.0
Self-ratings of olfactory function	Very bad	2	1.0
	Bad	0	0.0
	Moderate	15	7.5
	Good	106	53.0
	Very good	77	38.5

### QUESTIONNAIRES

Participants’ attitudes toward sense of smell, personality traits, and their demographics and self-ratings with respect to olfactory function were measured using self-administered questionnaires.

#### Attitudes toward sense of smell

To assess participants’ attitudes toward sense of smell, we used the “Importance of Olfaction Questionnaire” (IOQ) designed by Croy et al. (2010). The IOQ includes three subscales: “association,” “application,” and “consequence.” Each subscale is in turn composed of six questions to be answered with a 4-point category scale (1 = I totally disagree to 4 = I totally agree). The association-subscale indicates emotion, memory, and episode triggered by a sense of smell. The application-subscale reflects the extent to which people use sense of smell in their daily activities. Finally, the consequence-subscale reflects the extent to which people rely on sense of smell for daily decision-making. The additional subscale of “aggravation” developed for clinical applications (Croy et al., 2010) was not used because this study was designed for a general population.

#### Personality

Participants’ personality traits were assessed using the “Eysenck Personality Questionnaire-Revised” (EPQ-R; Eysenck et al., 1985). The EPQ-R, a 48-question self-reporting questionnaire, examines four major dimensions of personality trait: “psychoticism” (P: 12 questions), “extraversion” (E: 12 questions), “neuroticism” (N: 12 questions), and “lie-scale” (L: 12 questions). The psychoticism-subscale assesses behavior patterns used to characterize psychotic individuals or psychoses (Eysenck, 1997; Weiner and Craighead, 2010). The extraversion-subscale measures the extent to which individuals are sociable and active (Eysenck, 1997; Weiner and Craighead, 2010). The neuroticism-subscale assesses the extent to which individuals are predisposed to experience negative emotion (Eysenck, 1997; Weiner and Craighead, 2010). Finally, the lie-scale subscale reflects individuals’ socially conforming behaviors or their tendency to “fake good” (Weiner and Craighead, 2010).

### DEMOGRAPHICS AND SELF-RATINGS OF OLFACTORY FUNCTION

Participants’ demographics, such as gender, age, height, weight, ethnic background, annual household income, and education level, were assessed through a self-administered questionnaire. **Table 1** shows the participants’ demographic profiles. In addition, participants were asked to evaluate their own olfactory functions on a 5-point Likert scale ranging from 1 (very bad) to 5 (very good).

### DATA ANALYSIS

Data analysis was conducted using SPSS 21.0 for Windows^TM^ (IBM SPSS Inc., Chicago, IL, USA). Not all participants answered all questions (i.e., several participants did not complete all subscales; one for the association-subscale, two for the consequence-subscale, and two for the lie-scale subscale). Because the Shapiro–Wilk test (Shapiro and Wilk, 1965) revealed that the IOQ and the EPQ-R data were not normally distributed (**Table 2**), non-parametric statistical methods were used for data analysis. Mann–Whitney and Kruskal–Wallis tests were used to determine whether participants’ attitudes toward sense of smell varied as a function of demographic variables like gender, age, body mass index, annual household income, and education level. Spearman correlation coefficients were used to determine whether attitudes toward sense of smell were related to self-ratings of olfactory function. A relationship between participants’ personality traits and their attitudes toward sense of smell can be mediated by other factors that may possibly influence attitudes toward sense of smell, i.e., demographics and self-ratings of olfactory function (Croy et al., 2010; Seo et al., 2011). Therefore, to determine whether there is a relationship between personality traits and attitudes toward sense of smell, we used partial Spearman correlation analyses with treating potential factors to affect attitudes toward sense of smell as covariates. Calculating multiple correlations between personality traits and attitudes toward sense of smell can increase the risk of a type I error. That is, multiple correlation tests increase the probability of erroneously rejecting even one of the true null hypotheses (i.e., correlation coefficient is 0) when there is no significant correlation (Benjamini and Hochberg, 1995; Curtin and Schulz, 1998; Benjamini and Yekutieli, 2001). To avoid the risk of multiple correlation tests, the level of statistical significance of correlation coefficients was adjusted using Bonferroni’s correction (Curtin and Schulz, 1998). To keep the overall level of significance at 5% in this study, the level of significance for each correlation was divided by 12 (i.e., 4 dimensions of the EPQ-R by 3 subscales of the IOQ); the adjusted level of significance was set at *P* < 0.0042.

**Table 2:** Descriptive analysis results for ratings of personality traits and attitudes toward sense of smell.

						(*N* = 200 ^A^)
	Mean	Median	Standard deviation	Skewness	Kurtosis	Normality of data^B^
**Attitudes toward sense of smell^C^**
Association	19.0	19.0	2.4	-0.5	0.5	*W* = 0.97 (*P* < 0.001)
Application	17.6	18.0	2.9	-0.3	0.0	*W* = 0.98 (*P* = 0.012)
Consequence	17.4	18.0	2.6	-0.4	0.4	*W* = 0.98 (*P* = 0.001)
**Personality traits^D^**
Psychoticism	1.8	2.0	1.5	1.0	2.4	*W* = 0.89 (*P* < 0.001)
Extraversion	8.0	9.0	3.6	-0.6	-0.8	*W* = 0.90 (*P* < 0.001)
Neuroticism	4.5	4.5	3.2	0.3	-1.0	*W* = 0.95 (*P* < 0.001)
Lie-scale	4.7	4.0	2.6	0.3	-0.7

A Not all participants answered all questions (i.e., several participants did not complete all subscales; one for the association-subscale, two for the consequence-subscale, and two for the lie-scale subscale).

B Normality of data was determined by Shapiro–Wilk test (Shapiro and Wilk, 1965).

C Attitudes toward sense of smell were assessed by the Importance of Olfaction Questionnaire (IOQ; Croy etal., 2010).

D Personality traits were assessed by the Eysenck Personality Questionnaire-Revised (EPQ-R; Eysenck etal., 1985).

## RESULTS

**Table 2** presents the results of descriptive analysis for personality traits (EPQ-R) and attitudes toward sense of smell (IOQ). As previously mentioned, the data of the IOQ and the EPQ-R were not normally distributed (**Table 2**), so non-parametric statistical methods were used for data analysis. Before examining the relationship between participants’ personality traits and their attitudes toward sense of smell, potential factors that might possibly mediate the relationship, i.e., demographics and self-ratings of olfactory function, were determined.

### INFLUENCES OF DEMOGRAPHICS ON ATTITUDES TOWARD SENSE OF SMELL

**FIGURE 1:**
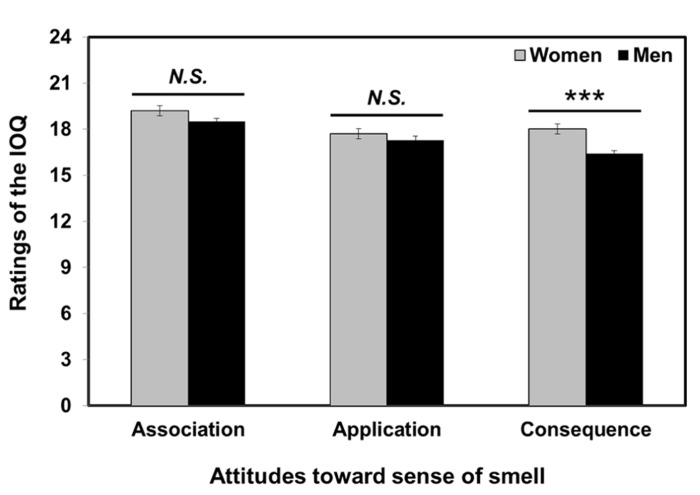
**Gender differences in the attitudes toward sense of smell.** Mann–Whitney *U*-tests revealed that women participants rated consequence-subscale of the IOQ (Importance of Olfaction Questionnaire) significantly higher than men participants. The asterisks (***) indicate significance at *P* < 0.001. Error bars represent standard error of the means.

Mann–Whitney *U*-tests revealed that women participants used olfactory cues for daily decision-making more often than men participants (*P* < 0.001), as shown in **Figure 1**. However, there was no significant gender-induced difference in the ratings of association-subscale (*P* = 0.15) and application-subscale (*P* = 0.23).

The Kruskal–Wallis tests found that the ratings of three subscales (i.e., “association,” “application,” and “consequence”) in the IOQ were not significantly different as a function of age groups (*P* > 0.05), body mass index (*P* > 0.05), education level (*P* > 0.05), and annual household income (*P* > 0.05).

### RELATIONSHIPS BETWEEN SELF-RATINGS OF OLFACTORY FUNCTION AND ATTITUDES TOWARD A SENSE OF SMELL

Spearman correlation analyses showed that participants’ self-ratings of olfactory function were positively correlated with the ratings of application-subscale (ρ_200_ = 0.17, *P* = 0.02) and consequence-subscale (ρ_198_ = 0.15, *P* = 0.03). For example, when participants judged their olfactory function to be more positive, they more frequently used their sense of smell in everyday life and for daily decision-making. Additionally, the self-ratings of olfactory function showed a marginally significant correlation with the ratings of association-subscale (ρ_199_ = 0.14, *P* = 0.05).

### RELATIONSHIPS BETWEEN PERSONALITY TRAITS AND ATTITUDES TOWARD A SENSE OF SMELL

As previously mentioned, we controlled potential factors that might mediate the relationship between personality traits and attitudes toward sense of smell. Based on these above results, participants’ gender and self-ratings of olfactory function were controlled in determining the relationship between their personality traits and attitudes toward sense of smell.

**Table 3** shows partial Spearman’s correlation coefficients (ρ) for the relationships between personality traits and attitudes toward a sense of smell. The ratings of consequence-subscale of the IOQ significantly correlated with the lie-scale scores at the Bonferroni-adjusted level of significance (ρ_191_ = 0.21, *P* = 0.0038). In other words, as participants showed socially conforming behaviors (e.g., fake good), they were more dependent on olfactory cues for daily decision-making.

**Table 3:** Partial Spearman correlation coefficients for the relationships between personality traits and attitudes toward sense of smell ^A^.

				(*N* = 200)
		Attitudes toward sense of smell^B^		
		Association	Application	Consequence
Personality traits^C^	Psychoticism	ρ_191_ = -0.06^N.S.^	ρ_191_ = -0.01^N.S.^	ρ_191_ = -0.001^N.S.^
	Extraversion	ρ_191_ = 0.10^N.S.^	ρ_191_ = 0.05^N.S.^	ρ_191_ = -0.08^N.S.^
	Neuroticism	ρ_191_ = 0.11^N.S.^	ρ_191_ = -0.01^N.S.^	ρ_191_ = 0.03^N.S.^
	Lie-scale	ρ_191_ = -0.02^N.S.^	ρ_191_ = 0.09^N.S.^	ρ_191_ = 0.21*

A When determining correlation between a dimension of the EPQ-R and a subscale of the IOQ, participants’ gender and self-ratings of olfactory function were treated as covariates.

B Attitudes toward sense of smell were assessed by the Importance of Olfaction Questionnaire (IOQ; Croy etal., 2010).

C Personality traits were assessed by the Eysenck Personality Questionnaire-Revised (EPQ-R; Eysenck etal., 1985).

*The level of significance for each correlation coefficient was adjusted using Bonferroni’s correction (Curtin and Schulz, 1998).*

The N.S. indicates no significance at *P* < 0.0042.The asterisk (*) indicates significance at *P* < 0.0042.

However, no other significant relationships among individual ratings of the IOQ and the EPQ-R were found at the Bonferroni-adjusted level of significance (*P* > 0.0042).

## DISCUSSION

### INFLUENCES OF DEMOGRAPHICS ON THE ATTITUDES TOWARD A SENSE OF SMELL

The current study shows gender-induced differences in attitudes toward sense of smell; compared to men, women participants reported that they use olfactory cues more often for daily decision-making. Although the gender difference was not apparent in all three subscales, our results are in agreement with earlier studies using the IOQ (Croy et al., 2010; Seo et al., 2011). Similarly, Croy et al. (2010) reported that the gender difference was obtained in the consequence-subscale, but not in the association- and application-subscales. More recently, Seo et al. (2011) reported that more women than men in four different regions: Mexico, Germany, Czech, and Korea, have attentive and positive attitudes toward sense of smell. For mate selection, men usually consider women’s visual appearance most important, while women tend to evaluate men’s body odors in determining superiority (Herz and Inzlicht, 2002; Havlicek et al., 2008). There are three possible explanations for gender-related differences in attitudes toward sense of smell. First, women’s superior olfactory performance (e.g., odor sensitivity, discrimination, identification; Doty et al., 1984; Hummel et al., 2007) may lead them to be more attentive and reactive to olfactory cues (Croy et al., 2010). Second, the proxemics theory of Hall (1966) might account for gender-induced attitudes toward sense of smell. Hall (1963) argued that people can establish interpersonal distances in eight different dimensions, including olfactory code. Generally, women stay closer to each other (i.e., smaller interpersonal distance) than men, which may provide them with more chances for judging other peoples’ body odors, identities, and emotional states (Seo et al., 2011). Finally, it should be noted that women participants in this study tended to be more neurotic and emotional than men participants (Mann–Whitney *U*-tests, *P* < 0.001). Considering the significant influence of neuroticism not only on olfactory performance, but also on attitudes toward sense of smell, women’s higher scores in neuroticism might result in more attentive attitudes to olfactory cues. However, because no significant correlation was found between ratings of neuroticism and the consequence-subscale exhibiting gender differences, further study should be conducted to support this assertion.

### RELATIONSHIPS BETWEEN SELF-RATINGS OF OLFACTORY FUNCTION AND ATTITUDES TOWARD SENSE OF SMELL

In this study, participants who judged their olfactory function more positively relied on olfactory cues in daily decision-making. These results are consistent with previous findings demonstrating a positive correlation between self-rating of olfactory sensitivity and general attitudes toward sense of smell (Seo et al., 2011). Self-assessment of olfactory function seems to be related to self-rating of nasal patency (Landis et al., 2003) or odor annoyance (Knaapila et al., 2008) rather than to olfactory perceptions such as odor sensitivity and discrimination (Landis et al., 2003). This result reflects the fact that individuals regarding their olfactory function more positively tend to be more attentive and reactive to the sense of smell regardless of olfactory sensitivity.

### RELATIONSHIPS BETWEEN PERSONALITY TRAITS AND ATTITUDES TOWARD SENSE OF SMELL

The above results demonstrate that gender and self-ratings of olfactory function may be associated with attitudes toward sense of smell. Therefore, factors such as gender and self-ratings of olfactory function were controlled as covariates when determining relationships between personality traits and attitudes toward sense of smell.

Previous research has focused on the idea that personality traits influence olfactory perceptions such as odor sensitivity (Koelega, 1970, 1994; Filsinger et al., 1987; Pause et al., 1998; Larsson et al., 2000; Croy et al., 2011b; Havlíček et al., 2012), odor intensity (Chen and Dalton, 2005), odor discrimination (Havlíček et al., 2012), odor identification (Larsson et al., 2000), and odor reaction time (Chen and Dalton, 2005; La Buissonnière-Ariza et al., 2013). Specifically, as people are more neurotic and anxious, they show better performance in detection (Pause et al., 1998; Chen and Dalton, 2005; Havlíček et al., 2012; La Buissonnière-Ariza et al., 2013), discrimination (Havlíček et al., 2012), and identification (Larsson et al., 2000) of olfactory cues. Based on previous research, it was expected that participants who scoring high in neuroticism (i.e., more anxious and emotional) would be prone to have more memory, episode, and emotion triggered by olfactory cues. According to Eysenck’s (1967) hypothesis, it is assumed that individuals high in neuroticism are more sensitive to emotional cues, especially aversive and negative stimuli, and this may be related to greater activation of the limbic system. Spearman correlation analysis showed that the scores of neuroticism-subscale significantly correlated with ratings of association-subscale of the IOQ at *P* < 0.05, but the significant relationship was not obtained at the Bonferroni-adjusted level of significance used in this study (*P* < 0.0042).

The lie-scale of the EPQ-R was designed to measure the tendency of respondents to lie or to fake effectively, thereby reflecting their acquiescence or conformity to social rules and pressures (Powell, 1977; Francis and Pearson, 1983). Interestingly, the current study demonstrated that participants scoring high in the lie-scale also showed high ratings in the consequence-subscale of the IOQ. In other words, individuals more constrained by social desirability (e.g., faking good) appear to rely more on olfactory cues when making daily-life decisions. A number of studies have elucidated that sense of smell is closely related to social communication and behavior (Wedekind et al., 1995; Gangestad and Thornhill, 1998; Chen and Haviland-Jones, 2000; Herz and Inzlicht, 2002; Olsson et al., 2006; Yamazaki and Beauchamp, 2007; Lundström et al., 2008; Prehn-Kristensen et al., 2009; Stevenson, 2010; Croy et al., 2011a, 2013; Mitro et al., 2012). Olfactory cues such as body odors reflect emotional state (Prehn-Kristensen et al., 2009; Croy et al., 2011a), individual identity (Olsson et al., 2006; Lundström et al., 2008), and sexual interests (Wedekind et al., 1995; Gangestad and Thornhill, 1998; Herz and Inzlicht, 2002; Croy et al., 2013; for review, see Yamazaki and Beauchamp, 2007; Stevenson, 2010). Olsson et al. (2006) asked participants to sniff the contents of five zip-lock bags containing both T-shirts worn by themselves, their friends, two strangers of opposite sex, and unworn T-shirts. They were then asked to identify the two shirts worn by themselves and their friends. Participants were able to determine not only their own T-shirts (51.6%), but also the T-shirts worn by their friends (38.7%). In addition, it is known that many people have the ability to recognize others’ emotional states such as happiness, fear, and anxiety (Chen and Haviland-Jones, 2000; Prehn-Kristensen et al., 2009) by smelling their body odors. A functional brain-imaging study demonstrated that body odors related to anxiety (produced during academic examination), in contrast to control group body odors (produced during bicycling), activated brain areas associated with the processing of social-anxiety information (e.g., fusiform gyrus) and the regulation of empathic feelings (e.g., insula, cingulate cortex, and precuneus). These findings reflect the fact that olfactory signals can play a key role in social communication in our society. Accordingly, it is thought that individuals more constrained by social desirability (i.e., high scores in lie-scale of the EPQ-R) tend to pay more attention to their own body odors, the better to provide positive and favorable impressions to others. In addition, they appear to judge other people’s identities, emotions, and personalities based on their body odors. In a similar vein, Croy et al. (2011b) demonstrated that agreeable participants, who tend to have greater concern for social harmony and cooperative nature (Rothmann and Coetzer, 2003), have higher sensitivities to odors. Furthermore, several studies have found that individuals with social deficits (e.g., autism and schizophrenia) have lower olfactory performances in areas like odor sensitivity (Dudova et al., 2011) and odor identification (Malaspina and Coleman, 2003). These findings support possible associations of social desirability (herein, lie-scale) not with only olfactory perceptions, but also with attitudes toward olfaction.

A plausible explanation for the relationship between smelling behavior and personality traits, especially social desirability, can be found in a neuroanatomical convergence of olfactory and emotional information in the limbic system, orbitofrontal cortex, insula, and anterior cingulate cortex (for a review, see Soudry et al., 2011). Functional brain-imaging studies have revealed that the limbic and paralimbic areas are involved in regulation of emotional and social desirability (Haas et al., 2010; Boehme et al., 2013) as well as in the processing of odor valence, odor memory, and odor-induced emotion (for review, see Gottfried, 2006; Soudry et al., 2011). Based on neuroanatomical convergence, it is to be expected that individuals who are faking good are vulnerable to emotional olfactory signals, possibly leading them to rely on olfactory cues for social communication and daily decision-making.

Since this research is a questionnaire-based study, a phenomenon known as the “extreme response style” (Hamilton, 1968; Greenleaf, 1992) should be noted. In other words, in questionnaire-based studies, regardless of specific item content, up to 30% of respondents are likely to consistently favor extreme response categories (Eid and Rauber, 2000; Austin et al., 2006; Naemi et al., 2009). Previous studies demonstrated that women and younger respondents tend to prefer extreme response categories compared to men and older respondents (Austin et al., 2006). In addition, respondents who scored high on extraversion and conscientiousness are likely to show a preference for extreme response categories (Austin et al., 2006). Because an extreme response style might result in a correlation between the ratings, the outcomes must be carefully interpreted. As seen in **Table 2**, both ratings of the EPQ-R and IOQ were highly skewed and, due to their non-normal distributions, non-parametric statistical methods were employed in this study, which might reduce the plausible overestimation caused by an extreme response style.

In summary, our findings provide empirical evidence that personality traits are related to attitudes toward sense of smell. Specifically, people constrained by social desirability (e.g., fake good) relied more on a sense of smell for daily decision-making. These findings provide better understanding of how personality traits are related to peoples’ attitudes toward sense of smell.

## Conflict of Interest Statement

The authors declare that the research was conducted in the absence of any commercial or financial relationships that could be construed as a potential conflict of interest.
